# Natural biocide cocktails: Combinatorial antibiotic effects of prodigiosin and biosurfactants

**DOI:** 10.1371/journal.pone.0200940

**Published:** 2018-07-19

**Authors:** Jennifer Hage-Hülsmann, Alexander Grünberger, Stephan Thies, Beatrix Santiago-Schübel, Andreas Sebastian Klein, Jörg Pietruszka, Dennis Binder, Fabienne Hilgers, Andreas Domröse, Thomas Drepper, Dietrich Kohlheyer, Karl-Erich Jaeger, Anita Loeschcke

**Affiliations:** 1 Institute of Molecular Enzyme Technology, Heinrich Heine University Düsseldorf, Forschungszentrum Jülich, Jülich, Germany; 2 IBG-1: Biotechnology, Forschungszentrum Jülich GmbH, Jülich, Germany; 3 Multiscale Bioengineering, Bielefeld University, Bielefeld, Germany; 4 Bioeconomy Science Center (BioSC), Forschungszentrum Jülich, Jülich, Germany; 5 Central Division of Analytical Chemistry ZEA-3: Analytik/Biospec, Forschungszentrum Jülich GmbH, Jülich, Germany; 6 Institute of Bioorganic Chemistry, Heinrich Heine University Düsseldorf, Forschungszentrum Jülich, Jülich, Germany; 7 Aachener Verfahrenstechnik (AVT.MSB), RWTH Aachen University, Aachen, Germany; Jamia Millia Islamia, INDIA

## Abstract

Bacterial secondary metabolites are naturally produced to prevail amongst competitors in a shared habitat and thus represent a valuable source for antibiotic discovery. The transformation of newly discovered antibiotic compounds into effective drugs often requires additional surfactant components for drug formulation. Nature may also provide blueprints in this respect: A cocktail of two compounds consisting of the antibacterial red pigment prodigiosin and the biosurfactant serrawettin W1 is naturally produced by the bacterium *Serratia marcescens*, which occurs in highly competitive habitats including soil. We show here a combinatorial antibacterial effect of these compounds, but also of prodigiosin mixed with other (bio)surfactants, against the soil-dwelling bacterium *Corynebacterium glutamicum* taken as a model target bacterium. Prodigiosin exerted a combinatorial inhibitory effect with all tested surfactants in a disk diffusion assay which was especially pronounced in combination with *N*-myristoyltyrosine. Minimal inhibitory and bactericidal concentrations (MIC and MBC) of the individual compounds were 2.56 μg/mL prodigiosin and 32 μg/mL *N*-myristoyltyrosine, and the MIC of prodigiosin was decreased by 3 orders of magnitude to 0.005 μg/mL in the presence of 16 μg/mL *N*-myristoyltyrosine, indicative of synergistic interaction. Investigation of bacterial survival revealed similar combinatorial effects; moreover, antagonistic effects were observed at higher compound concentrations. Finally, the investigation of microcolony formation under combined application of concentrations just below the MBC revealed heterogeneity of responses with cell death or delayed growth. In summary, this study describes the combinatorial antibacterial effects of microbial biomolecules, which may have ecological relevance by inhibiting cohabiting species, but shall furthermore inspire drug development in the combat of infectious disease.

## Introduction

Bacterial chemical defense mechanisms—developed to prevail amongst competitors in a shared habitat—have provided an ample source of effective antibiotics for clinical use [1], and continue to offer new promising drug candidates [2,3]. For many antimicrobial compounds, additional surfactant components are required in the formulation of an effective drug; their major function in pharmaceuticals is to improve the solubility of drugs, and enable penetration across biological interfaces [4,5]. Since the application of synthetic surfactants for pharmaceutical formulations evoked discussions regarding potentially toxic byproducts or issues regarding lack of biodegradability [6,7], novel bio-based solutions appear attractive. In nature, some bacteria living in highly competitive environments produce mixtures of antimicrobial and surface active compounds. Here, it may be suspected that the surface active compound likewise enhances effectivity of the antibiotic by improving delivery to the target. For instance, pigmented strains of the ubiquitous bacterium *Serratia marcescens* produce an antibiotic-surfactant mixture consisting of the red pigment prodigiosin and the antimicrobial biosurfactant serrawettin W1.

Prodigiosin is a bright red tripyrrole that belongs to the family of prodiginines. In *S*. *marcescens*, the prodigiosin biosynthetic pathway is encoded in a 21 kb gene cluster consisting of 14 *pig* genes [8]. Prodigiosin, which is produced from the precursors 2-octenal and proline in a complex bifurcated pathway, has several relevant properties such as immunosuppressive and anticancer activities toward different types of human cancer cells [9–11], as well as antimicrobial effects against Gram-positive and Gram-negative bacteria [12–15]. Several molecular mechanisms causing the antibiotic effects of prodigiosin are currently discussed, e.g., membrane potential alteration *via* anion symport [16], membrane damage [15], phototoxicity [17], and formation of reactive oxygen species (ROS) [18].

The symmetrical lipopeptide serrawettin W1, initially referred to as serratamolide, is composed of serine and β-hydroxyl fatty acids as the biosynthetic product of a non-ribosomal-peptide synthetase and produced by most of the colored *S*. *marcescens* strains [19–21]. Serrawettin W1 exhibits besides several interesting bioactivities, such as decreasing the viability of cancer cells [22,23], activity against oomycetes [24], and antimicrobial activity predominantly against Gram-positive bacteria [25,26] surfactant and wetting agent activity [27]. These activities appear typical for surface active bacterial metabolites such as surfactin, rhamnolipids and *N*-acyl amino acids [28,29].

The production of prodigiosin and serrawettin W1 in *S*. *marcescens* is described to be dependent to the same extent on several different factors like temperature, medium and growth phase [19,26,30]. Moreover, studies on the molecular level revealed a complex regulation network governing the concerted production of both metabolites [31–34]. This may suggest combinatorial effects of both compounds produced by *S*. *marcescens*.

Here, we report on the characterization of the combined antibacterial effects of isolated prodigiosin and serrawettin W1. We used the Gram-positive soil-dwelling *Corynebacterium glutamicum* as an exemplary target bacterium enabling demonstration of the compounds’ effects in a simple non-pathogenic showcase, which is moreover related to clinically concerning *Corynebacterium diphtheria* [35] and *Mycobacterium tuberculosis* [36]. Our results indicate enhanced combinatorial effects dependent on the ratio of prodigiosin and serrawettin W1. We further used this as a starting point to characterize mixtures of prodigiosin with other surface active compounds, including soil bacterial rhamnolipids and *N*-myristoyltyrosine, and observed enhanced combinatorial effects in all cases. The strong combinatorial effect of prodigiosin together with the less characterized *N*-myristoyltyrosine was further described focusing on the verge of bacteriostatic and bactericidal concentration ranges. Our findings suggest that naturally evolved compound cocktails may provide a suitable source for inspiring effective antibiotic development.

## Materials and methods

### Bacterial strains and cultivation conditions

*Pseudomonas putida* strains KT2440 [37], and pig-r2 [38], were cultivated in LB medium (Carl Roth®, Karlsruhe, Germany) [39] at 30°C, if not stated otherwise. *Escherichia coli* strain Tuner(DE3) (Merck, Darmstadt, Germany) was cultivated in LB medium at 37°C. *Corynebacterium glutamicum* (ATCC® 13032™) [40,41] was cultivated in LB medium at 30°C. *Serratia marcescens* DSM12481 was cultivated in LB medium at 30°C. Liquid cultures were incubated under continuous shaking at 130 rpm in a Multitron Standard incubation shaker (Infors AG, Bottmingen, Switzerland) in the dark. Antibiotics were added where appropriate in the following concentrations: Gentamycin, 25 μg/mL (*P*. *putida*), Kanamycin, 50 μg/mL (*P*. *putida* and *E*. *coli*).

### Applied antibiotic and surface active compounds

#### Prodigiosin

**Fig 1:**
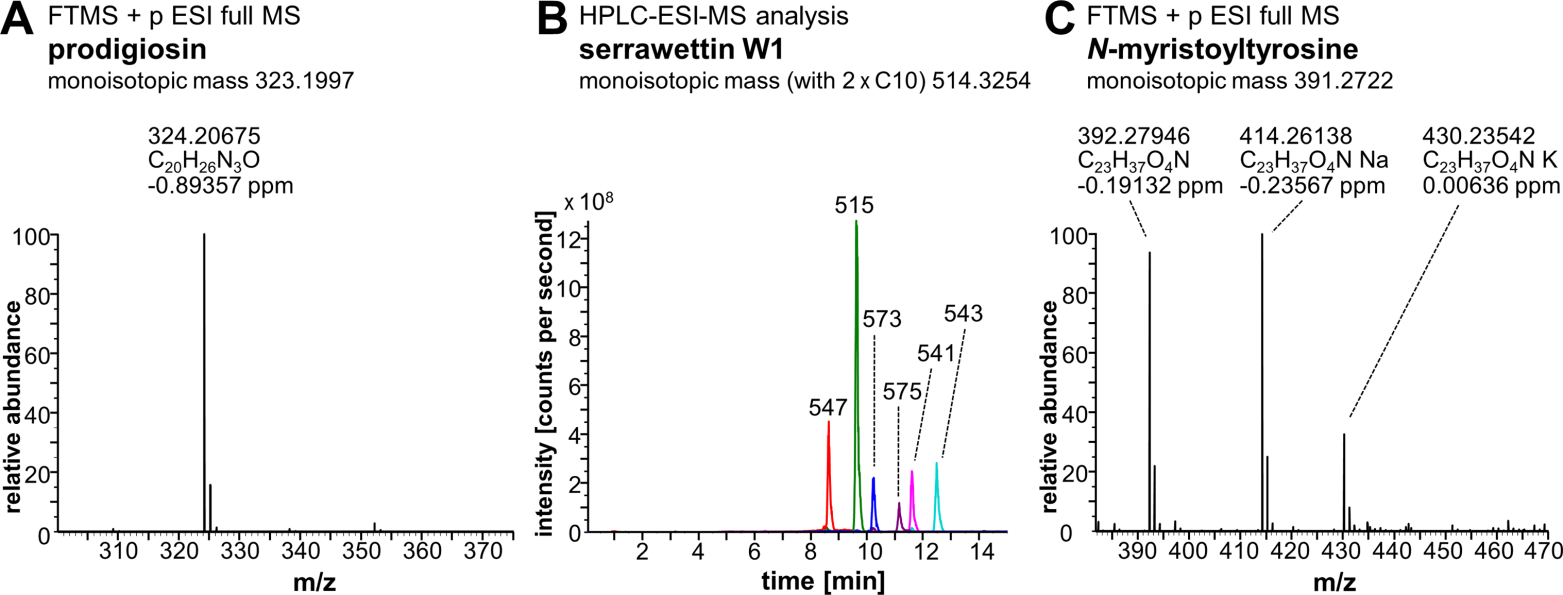
Mass spectrometry analysis of prodigiosin, serrawettin W1 and *N*-myristoyltyrosine. **(A)** FTICR-ESI-MS analysis of prodigiosin (M+H^+^)^+^ which was obtained by heterologous production in *P*. *putida*. The purified compound was solubilized in H_2_O/acetonitrile/formic acid (50/50/0.1%) and analyzed *via* infusion by FTICR-ESI-MS in the positive mode. **(B)** HPLC-ESI-MS analysis of serrawettin W1 which was obtained by heterologous production in *P*. *putida*. The extracted compound was analyzed by HPLC-ESI-MS. Detected nominal molecular masses (M+H^+^)^+^ of different serrawettin W1 species (congeners with different fatty acids varying in length and number of double bonds) are indicated in Da in overlayed extracted ion chromatograms (EICs) of m/z 547 with C10+C11+H_2_O (red), m/z 515 with C10+C10 (green), m/z 573 with C10+C13:1+H_2_O (blue), m/z 575 with C10+C13+H_2_O (violet), m/z 541 with C10+C12:1 (pink) and m/z 543 with C10+C12 (turquoise). **(C)** FTICR-ESI-MS analysis of *N*-myristoyltyrosine. Chemically synthesized *N*-myristoyltyrosine was solubilized in H_2_O/acetonitrile/formic acid (50/50/0.1%) and analyzed *via* infusion by FTICR-ESI-MS in the positive mode. Deviations of measured masses from monoisotopic masses of compounds (indicated in headlines) are given in ppm for FTICR-ESI-MS analyses.

Heterologous production of prodigiosin was established based on previously developed protocols using *P*. *putida* strain pig-r2 as production host for the expression of prodigiosin biosynthesis genes (*pig*) from *S*. *marcescens* [38]. Cells were grown in TB medium (Terrific-Broth modified; Carl Roth®, Karlsruhe, Germany) at 25°C and prodigiosin was recovered from the cultivation broth using polyurethane (PU) foam cubes. Prodigiosin was extracted from PU *via* Soxhlet extraction with diethyl ether and purified by two-fold flash column chromatography using dichlormethane and methanol (gradient: 0-1% (*v*/*v*) on silica gel 60 (particle size 0.040-0.063 mm, 230-240 mesh), yielding 65 mg prodigiosin per 1 L culture with a purity of 97% as determined by spectrophotometric analysis and application of previously determined extinction coefficient at 535 nm [38]. See **Fig 1** for mass calculation by Fourier transform ion cyclotron resonance mass spectrometry (FTICR-ESI-MS) analysis (LTQFT UltraTM, Thermo Fisher Scientific, Bremen, Germany).

#### Serrawettin W1

Serrawettin W1 was produced by heterologous biosynthesis *via* expression of the *swrW* gene from *S*. *marcescens*, essentially as described before [42], here using *P*. *putida* KT2440 as a production host. *P*. *putida* cells transformed with vector pVLT-swrW by electroporation [43] were used to inoculate LB medium and incubated at 30°C. Production was induced at an OD_580 nm_ of 0.5 with 0.4 mM IPTG and PU foam cubes were added for simultaneous product adsorption. After incubation for 18 h, PU foam was recovered, washed with water and extracted with ethanol. Dried extracts were re-dissolved and extracted with ethyl acetate and water, to remove polar components. Ethyl acetate extracts were pooled and dried to yield a crude light yellow-whitish serrawettin W1 extract, with a yield of 33.7 mg per 100 mL culture. A corresponding empty vector extract was produced analogously, yielding 1 mg of extract mass per 100 mL culture (about 3% of the weight of the serrawettin W1 extract). This may suggest that the serrawettin W1 extract contains about 97% of the compound. The mass difference between both extracts was thus considered when preparing appropriate samples of the empty vector control extract corresponding to the serrawettin W1 extract. HPLC-ESI-MS analysis was performed as described before [42] (**Fig 1**).

#### *N*-myristoyltyrosine

*N*-myristoyltyrosine was obtained by chemical synthesis, as described before [44]. The product was analyzed by FTICR-ESI-MS (**Fig 1**).

#### Rhamnolipids

A mixture of mono- and di-rhamnolipids was obtained as extract (90%) of *Pseudomonas aeruginosa* from Sigma-Aldrich®.

#### Synthetic surfactants

Tween 20, Triton X-100, and SDS were purchased from Carl Roth® (Karlsruhe, Germany).

For use in antibacterial assays, all compounds were dissolved in ethanol (p.a.).

### Disk diffusion assay

Based on previously established protocols [45], *C*. *glutamicum* or *S*. *marcescens* were cultivated in 10 mL LB without any antibiotics in 100 mL unbaffled Erlenmeyer flasks for 18 h at 30°C and constant shaking at 130 rpm. These freshly grown cells were used to prepare 1 mL saline solution (0.9% NaCl) with cells at an OD_580 nm_ of 0.3. A lawn of bacteria was generated on 120x120x15.8 mm LB plates (containing 1.5% (*w*/*v*) agar) by spreading using glass beads. For disk diffusion assays in a combination matrix, solutions of surfactants and prodigiosin (in ethanol, p.a.) were loaded on 0.75 mm thick round cellulose disks with 6 mm diameter (Carl Roth®, Karlsruhe, Germany) in 10 μL steps, followed by 5–10 min drying to reach 10, 25 or 50 μg of each compound per disk, alone and in combination. Pure ethanol without compounds was used at concentration = 0. Streptomycin which was used as a positive control was dissolved in water for application on disks. Dry loaded disks were transferred to agar plates with plated bacteria. Disk diffusion plates were incubated for 20 h at 30°C for lawn formation and photo-documented. For evaluation of *C*. *glutamicum* growth inhibition in compound combination matrices, inhibition zones were determined as clear areas in the lawn, and measured as diameter with subtracted disk size based on image files using the size of the disks (6 mm diameter) as a reference. For disk diffusion assays with *S*. *marcescens*, the pigment phenotype of bacteria in proximity to applied surfactants was photo-documented.

### Checkerboard growth inhibition and survival assay

Overnight cultures of *C*. *glutamicum* were inoculated in 10 mL LB in 100 mL unbaffled Erlenmeyer flasks and incubated at 30°C under agitation at 130 rpm. These were used to inoculate test cultures with an initial OD_580 nm_ of 0.05 in LB medium in Round Well Plates (m2p-labs, Baesweiler, Germany). Each well was filled with 776 μL bacterial culture and 24 μL antibiotic solution (i.e., 12 μL prodigiosin- plus 12 μL *N*-myristoyltyrosine-solution, both solved in ethanol) before cultivation. Control wells were supplemented with 24 μL pure ethanol. Two-fold serial dilutions of each compound, starting with 20.48 μg/mL prodigiosin and 32 μg/mL *N*-myristoyltyrosine were employed in a checkerboard matrix. Streptomycin was used as a positive control for reference. This antibiotic was added from stock solutions prepared in LB medium to cell suspensions in LB medium additionally supplemented with 3% ethanol to create the same setup as for prodigiosin and *N*-myristoyltyrosine. Round Well Plates were sealed with sterile breathable rayon film seals (VWR, Radnor, Pennsylvania, USA) and incubated for 20 h at 30°C and 600 rpm in an Eppendorf Thermomixer® (Eppendorf AG, Hamburg, Germany).

#### Determination of cell growth

Optical densities of washed cell cultures (see below) were measured using the microplate reader TECAN Infinite® M1000 PRO (Tecan Deutschland GmbH, Crailsheim, Germany) by turbidity measurements of samples in sterile 96-wells-F VWR® Tissue Culture Plates. To ensure linear correlation of turbidity measurements with cell density and avoid interference of the pigment prodigiosin, the wavelength of 650 nm was used which is beyond the absorption maximum of prodigiosin. Employing calibration, obtained values were converted to optical cell densities as measured in a standard spectral photometer with 1 cm path length. Bacterial growth was indicated by OD_650 nm_ values over 0.06. The minimal inhibitory concentrations (MICs) were determined as the lowest antibiotic concentration at which no growth was detected. Above this threshold, growth was further categorized as strongly (up to OD_650 nm_ = 1.00) or moderately impaired (up to OD_650 nm_ = 4.10) and indistinguishable from control (OD_650 nm_ ≥ 4.10). Based on MIC values, fractional inhibitory concentration (FIC) index values were determined for the classification in additive, synergistic or antagonistic effects. FIC index (FICI) is the sum of FIC values, which describe the relation of the required amount of a compound when combined with another to the required amount of a compound alone to inhibit bacterial growth: FICI = FIC prodigiosin [MIC (prodigiosin in combination) / MIC (prodigiosin)] + FIC *N*-myristoyltyrosine [MIC (*N*-myristoyltyrosine in combination) / MIC (*N*-myristoyltyrosine)].

#### Assessment of cell survival

Cells were washed by centrifugation and re-suspension in 800 μL fresh medium without antibiotics. After dilution (1:100), 3 μL samples were spotted onto LB agar plates which were incubated at 30°C for 20 h, and photo-documented. Untreated cells (0 μg/mL *N*-myristoyltyrosine and 0 μg/mL prodigiosin) were treated likewise as a control but further diluted (up to 10^−7^) and plated as 100 μL samples for CFU (colony forming unit) determination. Here, 1 mL *C*. *glutamicum* culture with OD_580 nm_ = 1 was found to correlate with 5.9 x 10^8^ CFUs. Minimal bactericidal concentrations (MBCs) were determined as the first antibiotic concentration at which 99.9% of bacteria (in relation to the untreated control) were killed, i.e., up to 55 colonies from 3 μL samples. Above this threshold, cell survival was further categorized as strongly impaired (just above 55 colonies, not countable), moderately impaired (scattered spots), and indistinguishable from control (fully grown spots).

### Microfluidic single-cell cultivation

For microfluidic single-cell cultivation, LB medium was filtrated using a sterile syringe polyethersulfone membrane filter with 0.2 μm pore size (VWR, Radnor, Pennsylvania, USA) to remove disturbing micro-particles. Experimental workflow including microfluidic chip fabrication [46,47] and microscopic live cell imaging setup and operation [48] was performed as previously described. To obtain fresh cell samples in a defined growth state, *C*. *glutamicum* was pre-cultured in 10 mL LB in 100 mL unbaffled Erlenmeyer flasks and incubated for 18 h at 30°C shaking at 130 rpm. Main cultures were inoculated from pre-cultures with an initial OD_580 nm_ between 0.02 and 0.04 and incubated under the same conditions. When cultures reached an OD_580 nm_ between 0.25 and 0.50, they were used for microchip inoculation, i.e., flushing of microchips with culture broth, aiming at capturing of single cells in monolayer growth chambers (see [49]). During the first phase of cultivation, cells were supplied with fresh LB at a constant perfusion rate of 200 nL/min and were allowed to adapt and perform one to two cell divisions during a 3 h period at 30°C. Afterwards, supplementation of LB containing prodigiosin (1 μg/mL) and *N*-myristoyltyrosine (15 μg/mL)–alone or in combination–was started. Compounds were dissolved in ethanol (p.a.) and added to the LB medium with a total volume of 3%. Thus, LB medium with 3% ethanol was used as a control. During this supplementation process, the perfusion rate was increased to 900 nL/min for 15 min until total exchange of medium. Thereafter, medium flow was stopped for the rest of the experiment. A minimum of 20 chambers was selected by random sampling for each condition (control, prodigiosin, *N*-myristoyltyrosine, and combination) to be monitored by time-lapse microscopy at 10 min intervals. Cultivation and imaging were performed for 20 h. Microcolony cell areas were determined using a tailor-made ImageJ plugin and plotted over time as a measure of growth [48]. Images of selected time points were extracted to depict cell growth of selected microcolonies.

## Results

### Combined effects of prodigiosin and surfactants

*S*. *marcescens* naturally produces the hydrophobic pigment prodigiosin in a mixture with a second antibiotic substance, namely the lipopeptide surfactant serrawettin W1. Here, we aimed to evaluate combinatorial effects of the two compounds *in vitro*, and investigate the effects of prodigiosin with other surface active substances. To this end, the antibacterial activities of compound mixtures were compared to the effects of single compounds on growth of the soil bacterium *C*. *glutamicum*, which constitutes a model system for Corynebacteriales [50,51], using a disk diffusion assay which represents an established method for initial straightforward determination of antibacterial effects [52].

**Fig 2:**
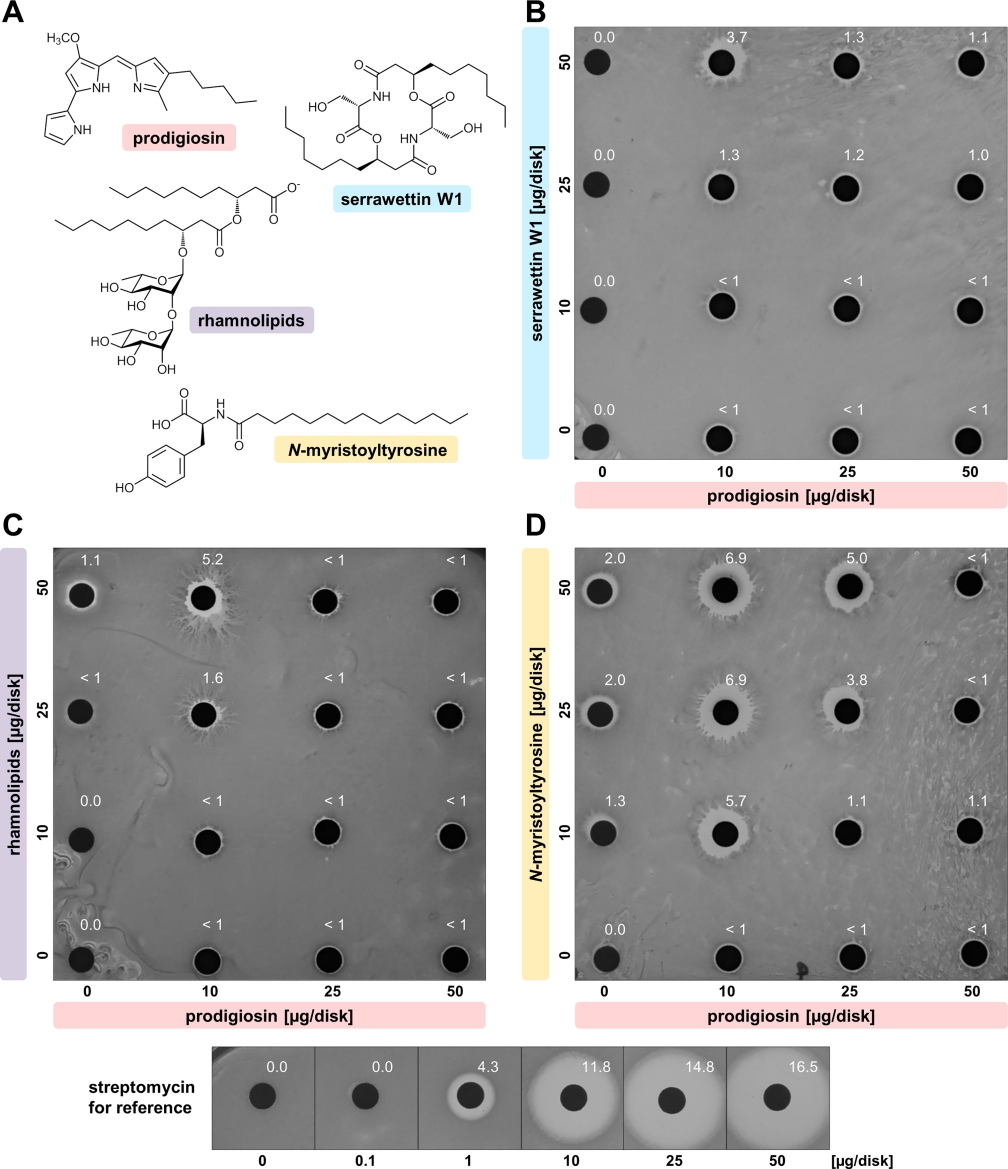
Combined effects of prodigiosin with different soil-bacterial surface active compounds on lawn-formation of *C*. *glutamicum*. **(A)** Compounds applied were the red pigment prodigiosin and the biosurfactant serrawettin W1, both produced by *S*. *marcescens*; rhamnolipid biosurfactants from *P*. *aeruginosa*; the biosurfactant *N*-myristoyltyrosine from an unknown enviromental bacterium. Combination matrices were implemented in disk diffusion assays on a lawn of *C*. *glutamicum* with compound combinations in series of 0, 10, 25, and 50 μg/disk: **(B)** Combination of naturally co-produced prodigiosin and serrawettin W1, **(C)** combination of prodigiosin and biosurfactant rhamnolipids, **(D)** combination of prodigiosin and biosurfactant *N*-myristoyltyrosine. Streptomycin was used as a positive control for reference. Shown photographed results are representative of duplicate experiments. Reach of inhibition zones from filter disks are indicated [mm].

As a first step, the effect of the naturally occurring mixture of *S*. *marcescens*, i.e., prodigiosin and serrawettin W1 (**Fig 2A**), was characterized. Both compounds were purified separately after heterologous production in *P*. *putida*, employing previously established protocols [38], and analyzed by mass spectrometry (**Fig 1A**, **Fig 1B**). Heterologously produced serrawettin W1 was found to be a mixture of congeners differing in the hydroxyl fatty acids, thus resembling the naturally occurring composition produced by *S*. *marcescens* [42,53]. To assess combinatorial effects, prodigiosin and serrawettin W1 were applied in a disk diffusion assay as a compound combination matrix using 0, 10, 25 and 50 μg of each compound per disk (**Fig 2B**). For reference, the anti-mycobacterial antibiotic streptomycin was used as a positive control.

The presence of the biosurfactant serrawettin W1 alone did not result in the formation of inhibition zones. In contrast, the antibacterial hydrophobic pigment prodigiosin evoked minor zones of inhibition with constant sizes (< 1 mm) at all concentrations tested. The combination of prodigiosin and serrawettin W1 generated a larger inhibition zone of 3.7 mm at the lowest amount of prodigiosin (10 μg/disk) together with the highest amount of serrawettin W1 (50 μg/disk). Any other combination of prodigiosin and serrawettin W1 only yielded minor inhibition zones (with maximal 1.3 mm reach), resembling inhibition zones generated by the single compound prodigiosin. To exclude that non-serrawettin W1 components of the extract affect the combinatorial effect, prodigiosin was combined in the same assay with extracts from *P*. *putida* cultures harboring the empty expression vector pVLT33 as a control. Here, no enhancement of prodigiosin-dependent inhibition zones could be observed (**S1 Fig**). The combination of prodigiosin and serrawettin W1 inhibited the growth of *C*. *glutamicum* in a concentration-dependent manner. This observation prompted us to test the effects of prodigiosin in combination with additional surfactants.

To investigate whether combinatorially enhanced effects of prodigiosin and serrawettin W1 are associated with the surface activity of serrawettin W1, prodigiosin was combined in the same disk diffusion assay with the frequently used synthetic surfactants Triton X-100 (octyl phenol ethoxylate), SDS (sodium dodecyl sulfate), and Tween 20 (polysorbate 20). Indeed, an enhanced combinatorial effect was observed in all cases (**S2 Fig**). Specifically, application of neither Triton X-100 nor Tween 20 alone resulted in the formation of inhibition zones, while higher amounts of SDS caused the formation of significant inhibition zones. When combined with low amounts of prodigiosin, Triton X-100 and, even more so, SDS evoked large inhibition zones. As observed with serrawettin W1, this effect was decreased with increasing amounts of prodigiosin. The application of Tween 20 similarly enhanced the inhibition compared to individual application of prodigiosin, but proved independent from the relative concentrations. These results cannot be related to physicochemical differences between the surfactants (cf. **S1 Table**). However, the common enhanced combinatorial effects with prodigiosin appear to be linked to the surface activity of the structurally different surfactant compounds.

To evaluate the potential of bio-based surfactants, which often exhibit advantageous biocompatibility and biodegradability [54], we investigated the effects of prodigiosin in combination with selected surface active compounds from other soil bacteria starting with rhamnolipids from *Pseudomonas aeruginosa* (**Fig 2A**). This bacterium synthesizes glycolipids consisting of L-rhamnose and β-hydroxyl fatty acids as a mixture of mono- and di-rhamnolipids [55]. These biosurfactants with antibacterial properties [56] are considered as an alternative to synthetic surfactants in diverse applications because of their favorable physicochemical properties and stability, as well as good biodegradability and low toxicity [57]. The compounds were applied in the disk diffusion assay combination matrix using 0, 10, 25 and 50 μg/disk prodigiosin and commercially obtained rhamnolipids (**Fig 2C**).

Here, a very similar result as obtained with serrawettin W1 was observed. Rhamnolipids only had a minor effect when applied alone (at 25 and 50 μg/disk) but the combination of prodigiosin and rhamnolipids produced a significantly enlarged inhibition zone of 5.2 mm at the lowest amount of prodigiosin (10 μg/disk) together with the highest amount of rhamnolipids (50 μg/disk). All other combinations generated inhibition zones resembling those evoked by the single compound prodigiosin.

The biosynthesis of surface active *N*-acylated amino acids was regularly found by metagenomic analysis of microorganisms in soil and other habitats [29,44,58]. Next, we tested *N*-myristoyltyrosine (**Fig 2A**) which we had previously identified, characterized and heterologously produced using *E*. *coli* as host [44], in combination with prodigiosin in the disk diffusion assay (**Fig 2D**).

Individually employed, the biosurfactant *N*-myristoyltyrosine produced small inhibition zones with increasing amounts from 10 μg/disk (1.3 mm) to 50 μg/disk (2.0 mm). Prodigiosin evoked minor zones of inhibition (< 1 mm) at all applied amounts as observed before (cf. **Fig 2B**, **Fig 2C**). In combination, these individual effects were altered remarkably. Any amount of *N*-myristoyltyrosine together with 10 μg/disk prodigiosin evoked significantly increased inhibition zones (5.7 to 6.9 mm), with strongest effects toward high amounts of *N*-myristoyltyrosine at 25 and 50 μg/disk. At 25 μg/disk prodigiosin, increased inhibition zones (3.8 to 5.0 mm) were only observed together with high amounts of *N*-myristoyltyrosine at 25 and 50 μg/disk. At 50 μg/disk prodigiosin in any combination with *N*-myristoyltyrosine, only minor inhibition zones (around 1 mm) were produced, representing a decrease as compared to the individual application of the biosurfactant.

Therefore, a combinatorial growth inhibition effect on *C*. *glutamicum*, highly dependent on the concentrations of the two compounds prodigiosin and *N*-myristoyltyrosine, could be determined. Similar to observations of the pigment’s activity in combination with serrawettin W1 or rhamnolipids, the combined effect appears to depend on the ratio of the two compounds, with lower amounts of prodigiosin in combination with high amounts of *N*-myristoyltyrosine evoking strongly enhanced effects, while higher amounts of prodigiosin diminished this effect or even showed antagonistic results. It is worth mentioning that in comparison to effects evoked by streptomycin, which was used as positive control, observed inhibition zones were relatively small.

In summary, our initial experiments with disk diffusion assays revealed combinatorial antibacterial effects of prodigiosin and surfactants and showed that the combination of the pigment with *N*-myristoyltyrosine yielded most pronounced effects among the biosurfactants which was thus defined as the most interesting combination for further characterization. Further, the disk diffusion assay entails some limitations as the readout is dependent on the effective diffusion of compounds out of the disks into the agar, which is hampered in case of hydrophobic compounds in comparison to more polar compounds like streptomycin. Therefore, it remains unclear whether the surfactants promote enhanced inhibitory effects by facilitating the distribution of the hydrophobic prodigiosin on the surface of the agar plate or by acting directly on *C*. *glutamicum* cells, which, in concert with prodigiosin-mediated effects, may lead to stronger inhibitory effects. Consequently, we analyzed the combinatorial effects of prodigiosin and the biosurfactant *N*-myristoyltyrosine in more detail with further methods.

### Growth inhibition and survival of *C*. *glutamicum* exposed to prodigiosin and *N*-myristoyltyrosine

The minimal inhibitory concentration (MIC) constitutes a commonly applied parameter for the description of antibacterial effects. Thus, MICs were determined for prodigiosin and *N*-myristoyltyrosine, individually and in combination, which allowed the determination of the corresponding fractional inhibitory concentration (FIC) index to assess combinatorial effects.

**Fig 3:**
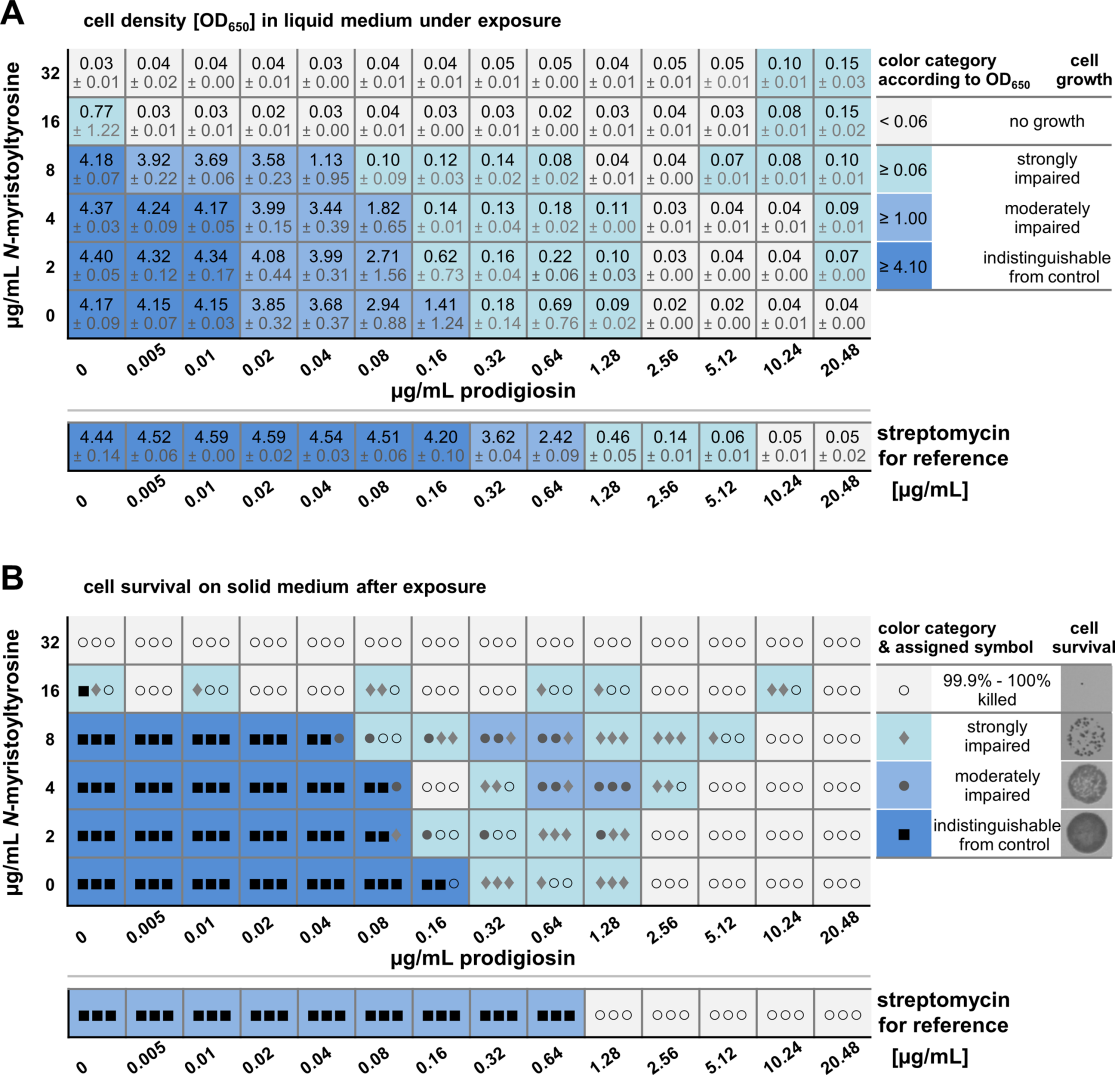
Growth and survival of *C*. *glutamicum* under combined exposure to prodigiosin and *N*-myristoyltyrosine. Liquid cultures (volume 800 μL) of *C*. *glutamicum* were supplemented with antibiotic compounds in a checkerboard matrix individually and in combinations in two-fold dilutions. The antibiotic streptomycin was individually applied for reference. **(A)** Turbidities of cultures were measured at 650 nm after incubation for 20 h at 30°C and constant agitation. Grey: Turbidities up to 0.06, defined as no growth. Different shades of blue: Strongly impaired growth with turbidities from 0.06 to 1.00, moderately impaired growth with turbidities from 1.00 to 4.10, and unimpaired growth with turbidities above 4.10. Values represent mean results from three independent measurements and the respective standard deviation. **(B)** Grown cells were washed and diluted samples spotted onto solid medium without antibiotics for incubation at 30°C for 20 h. Colony formation was evaluated by assignment of categories according to cell survival: killed (grey, empty circle), strongly impaired (light-blue, filled diamond), moderately impaired (blue, filled circle), and fully grown like the control (dark-blue, filled squares). Symbols in the table represent respective results from three independent experiments.

*C*. *glutamicum* was cultivated in the presence of different concentrations of prodigiosin and *N*-myristoyltyrosine in a small volume (800 μL). The antimicrobial pigment and surfactant were supplemented in a checkerboard matrix individually and in combination in two-fold dilutions starting from 20.48 μg/mL and 32 μg/mL, respectively, to 0.005 μg/mL and 2 μg/mL. MICs were determined as the minimal compound concentration required to inhibit bacterial growth under the here applied experimental setup. Bacterial growth was evaluated *via* turbidity measurements after 20 h of cultivation (**Fig 3A**). Bacterial growth was indicated by OD_650 nm_ values between 0.06 and 4.40 (**Fig 3A**, different shades of blue), while values up to 0.06 indicated no growth (**Fig 3A**, grey). Streptomycin was employed as a positive control for reference.

When applied individually, both compounds were inhibitory and MIC values of prodigiosin and *N*-myristoyltyrosine were thus defined as 2.56 mg/mL and 32 mg/mL, respectively. These values are in a similar range as the MIC of the positive control streptomycin which exerted strong inhibition at ≥ 1.28 μg/mL and completely inhibited bacterial growth at 10.24 μg/mL. In combination, MIC values of both compounds declined significantly, namely to 0.005 μg/mL prodigiosin with 16 μg/mL *N*-myristoyltyrosine, or to 1.28 μg/mL prodigiosin with 8 μg/mL *N*-myristoyltyrosine. Moreover, in presence of prodigiosin in the sub-MIC concentration range of 0.32 to 1.28 μg/mL, bacterial growth was apparently possible but clearly inhibited with optical densities between 0.09 and 0.69. The occurrence of such an intermediate response at certain concentrations is not unexpected but typically observed in similar checkerboard experiments with other antibiotics [59]. Together with 2 to 8 μg/mL *N*-myristoyltyrosine, this range was enlarged to lower prodigiosin concentrations of 0.16 or 0.08 μg/mL prodigiosin. Interestingly, the combined application of both compounds at high concentrations (5.12 to 20.48 μg/mL prodigiosin and 8 to 32 μg/mL *N*-myristoyltyrosine) appeared to favor slight bacterial growth with optical densities between 0.06 and 0.15. Therefore, both an increase and a decrease of antibiotic effects were observed depending on the respective concentrations.

In order to classify the combinatorial effects regarding synergy, additivity or antagonism, we determined FIC values (fractional inhibitory concentration) *via* division of MIC values at combined application by MIC values of individually applied compounds. The FIC thus describes the relation of the amount of a compound when combined with another, to the amount of a compound alone that is required to inhibit bacterial growth. The decisive FIC index (also FICI) was calculated as the sum of individual FIC values (**Table 1**). Following the most common categorization [60,61], a synergistic effect exists at a FIC index ≤ 0.5, indifference is defined by 0.5 < FICI ≤ 4, and an antagonistic effect is classified by a FICI value > 4. This categorization is based on the concept that a mean 4-fold enhancement of the antibiotic effect of both compounds is considered as significant synergism, and a mean 4-fold reduction of their effect as true antagonism. However, diverse studies argue that FIC index values up to 1 (corresponding to a mean 2-fold enhancement) are indicative of synergy, and values above 1.25 (corresponding to a mean 1.6-fold reduction) indicate antagonism [62]. Here, two FIC indices could be determined at lowered combined MICs. Results revealed individual FIC indices of 0.002 and 0.5 for one specific combination of 0.005 μg/mL prodigiosin and 16 μg/mL *N*-myristoyltyrosine, respectively, and a resulting FIC index of 0.502, which points toward a synergistic effect of prodigiosin and *N*-myristoyltyrosine. Another combination of 1.28 μg/mL prodigiosin and 8 μg/mL *N*-myristoyltyrosine was characterized by individual FIC indices of 0.5 and 0.25 of prodigiosin and *N*-myristoyltyrosine, respectively, and a corresponding FIC index of 0.75. Therefore, a clear tendency toward a synergistic interaction of the two compounds could be determined at the named concentrations. Moreover, observations of minimal growth at higher compound concentrations might indicate additional antagonistic effects, here not captured by FIC index determination.

**Table 1:** MICs and FIC indices describing the combined effects of prodigiosin and *N*-myristoyltyrosine on *C*. *glutamicum*. Individual MICs of compounds, as well as MICs at two combinations (combi I: 0.005 μg/mL prodigiosin and 16 μg/mL *N*-myristoyltyrosine, combi II: 1.28 μg/mL prodigiosin and 8 μg/mL *N*-myristoyltyrosine), which exhibited enhanced inhibitory effects, were used to calculate FIC and FIC index values.

	alone	combi I			combi II		
	MIC [μg/mL]	MIC [μg/mL]	FIC*	FIC index**	MIC [μg/mL]	FIC*	FIC index**
**prodigiosin**	2.56	0.005	0.002	0.502 (minor synergy)***	1.28	0.5	0.75 (minor synergy)***
***N*-myristoyl-tyrosine**	32	16	0.5		8	0.25	

* fractional inhibitory concentration, FIC = MIC_combi_ / MIC_alone_

** FIC index = FIC_prodigiosin_ + FIC_*N*-myristoyltyrosine_

*** type of interaction (categorization *via* FICI values: ≤ 0.5—synergy; 0.5-1—minor synergy; 1-1.25—indifference; ≥ 1.25-4—minor antagonism; ≥ 4—antagonism)

In order to evaluate not only growth inhibition but also bactericidal effects of prodigiosin and *N*-myristoyltyrosine, *C*. *glutamicum* cells were washed after incubation in media with different antibiotic concentrations and combinations, sampled for incubation on agar plates in the absence of antibiotics, and subsequently viability was determined documenting colony formation (**Fig 3B**). Bacterial survival was indicated by significant colony formation (**Fig 3B**, different shades of blue and filled symbols), while bactericidal concentrations were defined as killing ≥ 99.9% of bacteria (**Fig 3B**, grey and empty circles).

Minimal bactericidal concentrations (MBCs) of prodigiosin and *N*-myristoyltyrosine were found to be 2.56 μg/mL and 32 μg/mL, respectively, corresponding to previously determined MICs. Likewise, prodigiosin alone significantly reduced cell survival at 0.32 to 1.28 μg/mL, and, when combined with 2 to 8 μg/mL *N*-myristoyltyrosine, this effect was extended to even lower prodigiosin concentrations of 0.08 or 0.16 μg/mL prodigiosin (**Fig 3B**, light-blue). Streptomycin-exposed cells, which were used as a positive control, were unable to survive at 1.28 μg/mL. The observation of a lower MBC than MIC for the antibiotic seems coherent with its mechanism of action as streptomycin interferes with ribosomal protein biosynthesis which leads to errors during translation, synthesis of defective proteins, inhibition of protein synthesis and ultimately cell death. Apparently, initial cell divisions can be completed and become apparent as turbidity in the MIC determination, but these cells seem to have entered a lethal state of impairment.

Combined with 16 μg/mL *N*-myristoyltyrosine, prodigiosin showed enhanced bactericidal effects from 0.005 to 0.32 μg/mL, although here, fluctuating results were obtained over the concentration range as depicted by different symbols indicating the results from triplicate experiments. At the same time, under the influence of *N*-myristoyltyrosine at concentrations of 4 to 16 μg/mL, also a tendency to slight bacterial growth at prodigiosin concentrations from 0.64 to 10.24 μg/mL, notably both below and above its individual MBC was observed, again pointing to possible antagonistic combinatorial effects. Finally, very high concentrations of 20.48 μg/mL prodigiosin and 32 μg/mL *N*-myristoyltyrosine, which previously resulted in slightly increased cell densities during the investigation of MICs, were shown to fully kill bacteria (**Fig 3B**). Under these conditions, bacteria are apparently able to perform initial cell divisions that are, however, limited to only few events and cannot support survival.

### Combined effects of prodigiosin and *N*-myristoyltyrosine at the single cell level

**Fig 4:**
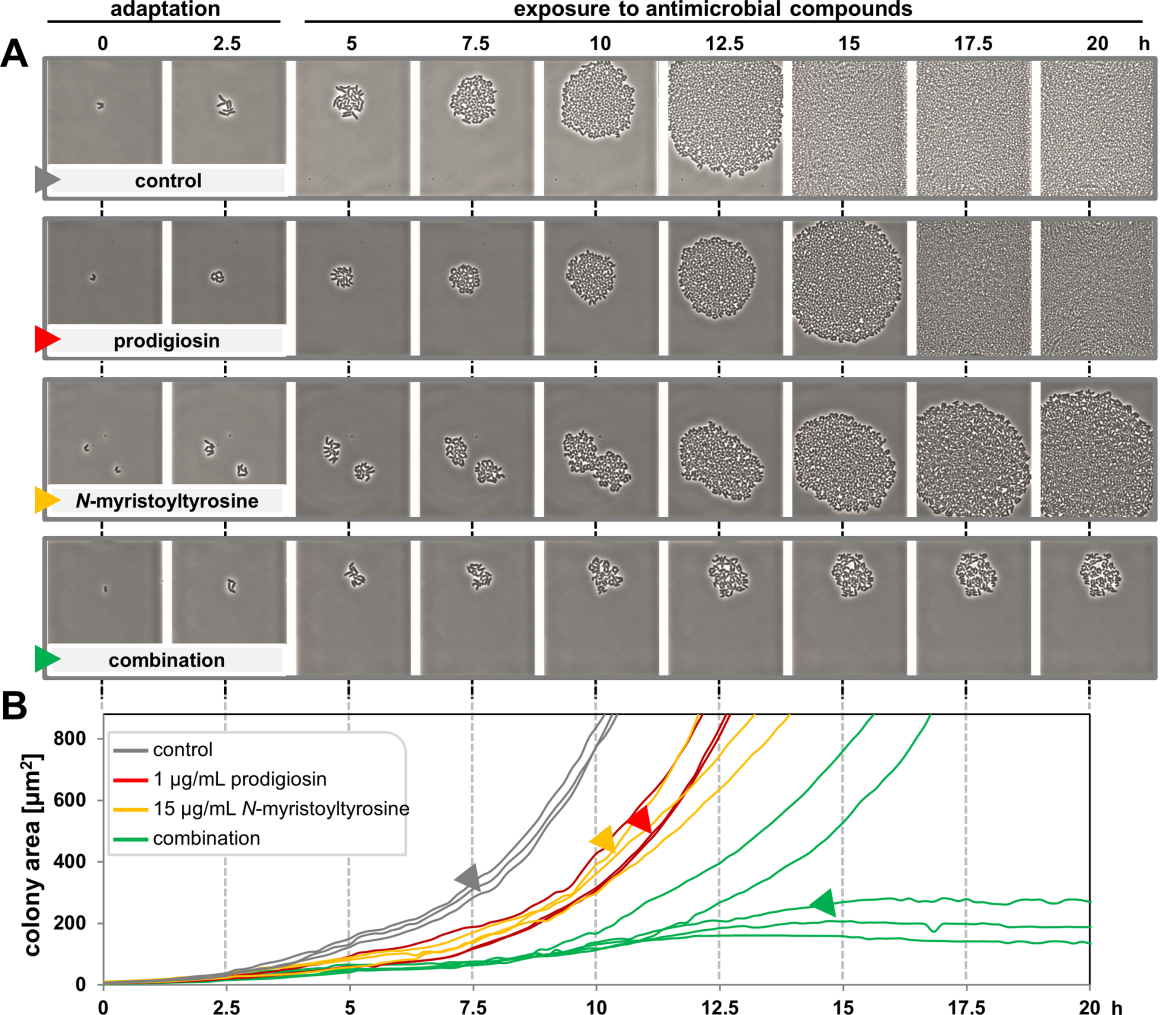
Microcolony formation by *C*. *glutamicum* in the presence of prodigiosin and *N*-myristoyltyrosine. Growth chambers were inoculated with bacteria at time point “0 h”. After 3 h, cells were exposed to medium supplemented with antibiotics, individually or in combination, and the control was supplemented with 3% ethanol. Microcolony formation was documented by time-lapse microscopy over 20 h with 10 min intervals. **(A)** Cell growth is depicted for individual microcolonies in images extracted from selected time points (see image series). **(B)** The development of colony area over time is shown as a measure of bacterial growth for different microcolonies. Data was collected in three independent experiments, monitoring a total of 60 microcolonies per condition. For conditions evoking a uniform response, three representative microcolonies were selected; for exposure to both compounds, five microcolonies were selected to represent differential response. Curves marked with colored arrows correspond to depicted image data.

To gain further insights into antibiotic effects at the single cell level, *C*. *glutamicum* was subjected to microfluidic single cell cultivation [48,63] in the presence of prodigiosin, *N*-myristoyltyrosine and their combination. Live cell imaging was used to analyze if the observed impaired growth resulted from homogeneous effects exerted on all cells or represents a result averaged from differentially responding single cells. To this end, prodigiosin and *N*-myristoyltyrosine were applied at concentrations of 1 μg/mL and 15 μg/mL, respectively, which were previously determined to exhibit inhibitory effects on cell growth without effective killing. After inoculation and 3 h incubation allowing cells to adapt and perform initial divisions, cells were perfused with medium supplemented with antibiotics for 15 min. Thereafter, the flow was stopped until the end of the experiment, creating a batch cultivation comparable to previously published experiments [64]. Since compounds were dissolved in ethanol, LB medium supplemented with 3% ethanol served as a control. Image data were extracted from selected time points to display microcolony development over the course of the experiment (**Fig 4A**). Time point “0 h” represents cells after their inoculation in the microfluidic cultivation system. Between 12.5 and 15 h, control chambers were filled with *C*. *glutamicum* cells due to continuous cell division. Microcolonies developing in the presence of prodigiosin or *N*-myristoyltyrosine were only slightly impaired in growth, whereas those supplemented with the compound cocktail of 1 μg/mL prodigiosin and 15 μg/mL *N*-myristoyltyrosine were strongly inhibited. Here, chambers were not filled at the end of the experiment, i.e., cell growth had ceased. Individual cells underwent phenotypic changes forming unusual cell shapes and even appeared to loose cell integrity and burst during online monitored microcultivation (see **S1 Video**, **S3 Fig**).

However, inspection of all monitored chamber showed that cell behavior in the presence of two antibiotic compounds varied between different microcolonies. In order to more broadly depict bacterial responses to antibiotic exposure accordingly, the colony area was monitored as a measure of growth over time in several chambers at each condition (**Fig 4B**). In the control experiment, microcolonies exhibited constant, fast growth. Under exposure to individual compounds, likewise constant results were obtained, with microcolony growth showing a prolonged lag phase (by about 2.5 h) before starting growth with similar kinetics as the control. However, treated with the cocktail of prodigiosin and *N*-myristoyltyrosine at the chosen concentrations, microcolonies showed highly differential behavior, ranging from growth after a significantly prolonged lag phase to complete growth arrest, which occurred in about 55% of monitored chambers.

Growth of a subpopulation of cells might be the result of resistance caused by an early mutation, possibly induced by the applied compounds, e.g., by ROS formation [65], or attributed to non-heritable physiological adaptions [66]. To distinguish between both phenomena, a re-exposition assay was conducted. To this end, *C*. *glutamicum* was treated with a combination of 1 μg/mL prodigiosin and 15 μg/mL *N*-myristoyltyrosine in small scale liquid cultivation as described for checkerboard experiments, and surviving clones obtained in subsequent cultivation on agar plates without antibiotics were re-subjected to exposure in liquid cultivation with individual antibiotics at MBC concentrations. Assessment of survival of these cells in the absence of antibiotics on agar plates revealed that cells were effectively killed, thus showing unchanged susceptibility toward bactericidal concentrations of the compounds (**S4 Fig**). Therefore, the occurrence of mutations conveying complete resistance is unlikely; instead, physiological adaptions in individual cells seem to be more reasonable.

In summary, a strong combined antibacterial effect of prodigiosin and *N*-myristoyltyrosine could be demonstrated also at the single cell level. Moreover, a differential development of individual microcolonies was observed in presence of concentrations that were previously determined as impairing growth but not fully killing. This points toward a heterogeneity in cellular response and indicates that the presence of a few pre-adapted cells may have a significant influence on the overall growth behavior of the population, which is typically evaluated in MIC determination. The phenomenon may further account for the observed fluctuating numbers and scattered occurrence of surviving cells in viability assays.

The combination of methods and compounds used here for the analysis of antimicrobial effects on *C*. *glutamicum* resulted in the following emerging picture: For the natural combination of prodigiosin and serrawettin W1, as well as the deduced artificial cocktails including prodigiosin and *N*-myristoyltyrosine, combinatorial impairment of bacterial growth was clearly detectable in disk diffusion assays although inhibition zones were rather small at relatively high compound dosage. For the latter combination, we used liquid medium-based checkerboard assays for further characterization and determined MICs and MBCs of both compounds alone in the μg/mL range and in combination in the μg to ng/mL range, finding synergistic features in growth inhibition and both synergistic and antagonistic features in bacterial killing. In addition, analysis on single-cell level indicated that killing efficacy at intermediate concentrations may be prone to cell heterogeneity among the populations. The differential responses of single cells during exposure and scattered appearance of surviving cells after exposure, without acquisition of resistance, suggest individual adaptation processes in *C*. *glutamicum* in this concentration range.

## Discussion

This study demonstrated that isolated prodigiosin and serrawettin W1, two antimicrobial compounds concertedly produced by *S*. *marcescens*, exert combinatorial enhanced effects *in vitro* against *C*. *glutamicum* in a concentration-dependent manner. Even more pronounced concentration-dependent effects were observed for a combination of prodigiosin and *N*-myristoyltyrosine.

Synergistic activities of combinations of diverse antibiotics with surface active antimicrobial compounds have been reported various times [67–73]. The ecological function of the simultaneous production of combinations of bioactive metabolite by a single microbe may be an increase in effectiveness [74–80]. If not to kill competitors completely, the function of antibiotic biosynthesis may be to create colonization advantages for the producing organisms by inhibiting or delaying the growth of competing microorganisms. It is interesting to note that especially in the soil niche, a number of bacteria were found to be genetically equipped for the production of several bioactive substances (e.g., *Streptomyces*, *Pseudomonas* and *Serratia* species [77,81,82]), including the producers of the here investigated serrawettin W1 and prodigiosin (*S*. *marcescens*) [83,84], or rhamnolipids (*P*. *aeruginosa*) [67,85]. The soil habitat, as a complex and highly competitive environment, may provide a particularly pronounced evolutionary pressure toward the development of a versatile arsenal of chemical defense mechanisms. Our results prompt consideration of two specific hypotheses regarding prodigiosin co-functioning with surfactants:

Possibly, the combination of prodigiosin with a surface active metabolite represents a general natural scheme for enhanced bioactivity as it appears to have developed several times with structurally different surfactant components. First, the present study shows that the mixture of isolated prodigiosin and the lipopeptide serrawettin W1, as it occurs in *S*. *marcescens* DSM12481, exhibits increased antibiotic activity. Furthermore, a previous study suggested that prodigiosin produced by *Serratia* sp. ATCC 39006 exerts antibiotic activity only in combination with a biosurfactant produced by this strain [70]. There, the biosurfactant was assumed to be a 3-(3-hydroxyalkanoyloxy)alkanoic acid (HAA)-related compound, which was deduced from the homology of a protein associated with surfactant production to *P*. *aeruginosa* RhlA, which catalyzes the formation of HAA as the first step in rhamnolipid biosynthesis. Serrawettin W1 biosynthesis does probably not occur in this particular strain because by analysis of the available sequence data and associated protein sequences with BLAST P and antiSmash [86,87] we were unable to identify a protein resembling serrawettin synthase [88]. It may further be assumed that glycolipids similar to rhamnolipid or rubiwettin might be produced by the strain since its genome also contains a homolog to rhamnosyl transferase RhlB from *P*. *aeruginosa* (see **S5 Fig**). Finally, a prodigiosin synthesizing strain was recently described to be unable to produce serrawettin W1, but remarkably could produce serrawettin W2, a structurally divergent, more complex cyclic lipopeptide [89]. Here, first hints suggest a co-regulation of biosynthesis pathways [90], but future research is needed to elucidate if combinatorial effects can also be found in this case.

Furthermore, in densely populated habitats like soil, combinatorial effects of compounds not only secreted by a single bacterial strain, but also of different cellular origin may occur. This way, inhabitants may benefit from the presence of compounds produced by other microbes. Interestingly, prodigiosin biosynthesis in its natural producer *S*. *marcescens* is known to be enhanced by the presence of surfactants, in particular by SDS [91]. Intriguingly, all surfactants tested in this study– with the exception of Tween 20 –not only inhibited *C*. *glutamicum* growth when combined with prodigiosin, but also appear to induce enhanced prodigiosin biosynthesis in the natural producer *S*. *marcescens* (**S6 Fig**). It is unknown whether this might be due to physicochemical processes, like an *in situ* extraction of the compound from *S*. *marcescens* membranes into surfactant micelles influencing the biosynthetic reaction equilibrium toward increased production as previously suggested [92], or due to regulatory processes increasing prodigiosin production in response to a surfactant. Nevertheless, it may be speculated that *S*. *marcescens* might benefit from the presence of other surfactants in soil by their combinatorial antibacterial effects together with *S*. *marcescens*-derived prodigiosin.

A prerequisite for clinical application is the understanding of the antibiotic mechanisms of compounds or compound combinations. The enhanced antibacterial activity of prodigiosin together with surface active compounds could be a result of different underlying mechanisms. The antibacterial activity of prodigiosin has been described several times, with reported MICs in the concentration range of 1 to 8 μg/mL against different *Staphylococcus aureus* and *Bacillus subtilis* strains [12,13], generally matching the MIC of 2.56 μg/mL against Gram-positive *C*. *glutamicum* as determined in this study. We furthermore show here that in combination with 16 μg/mL *N*-myristoyltyrosine, 0.005 μg/mL prodigiosin fully inhibits growth of *C*. *glutamicum*. Diverse mechanisms inducing bacterial programmed cell death, including DNA intercalation, have been assigned to the pigment’s antibiotic activity [18]. For activity against *Bacillus spec*. in particular, the disturbance of membrane integrity and induction of bacterial autolysis, as a result of proton/Cl^-^ symport *via* the cell membrane was reported [13]. In line with that, we corroborate here bactericidal activity of prodigiosin and observed that *C*. *glutamicum* cells treated with prodigiosin and *N*-myristoyltyrosine displayed compromised cell integrity.

In contrast, the antibacterial activities of here employed biosurfactants have not been discussed on a detailed mechanistic level. Serrawettin W1 and *N*-myristoyltyrosine especially affect Gram-positive bacteria [25,26,44], a surfactant-typical phenomenon assigned to the specific architectures of the cell envelopes. Since we show here that a range of structurally very diverse surfactants evokes enhanced inhibition of *C*. *glutamicum* together with prodigiosin, as compared to individual application, the common surface active properties appear central for this function as discussed above. *Via* their surface active properties they presumably destabilize bacterial lipid membranes and may enhance solubility, and therefore, the bioavailability, i.e., entrance into the cell membrane or cell interior, of hydrophobic prodigiosin. The here observed different combinatorial effects dependent on the ratio of surfactant to prodigiosin may be explained by concentration-dependent differences in the mode of surfactant assembly (e.g., in monolayers, bilayers, micelles, vesicles), and may be modulated by different concentrations of prodigiosin. In addition to surface activity, further differential activities of the surfactants cannot be excluded. Notably, it is known that surfactants can function highly specifically for a given organism, from inhibiting to enabling developmental processes, regardless of their shared ability to reduce surface tension [93]. In the context of drug development and clinical application, surfactants should not be mistaken as additives merely relevant for the manufacturing process of antibiotics. They should be appreciated also as bioactive compounds with valuable functions i.e., exhibiting innate antimicrobial properties and enhancing antibiotic effectivity [94,95]. Examples for the latter include improving e.g. antibiotic-based biofilm disruption relevant for wound treatment [96] or antibiotic delivery and bioavailability in otherwise difficult-to-target areas [97,98]. Interestingly, here investigated *N*-myristoyltyrosine promoted an enhanced effect of the anti-mycobacterial antibiotic streptomycin against *C*. *glutamicum* in an initial evaluation (**S7 Fig**).

Besides dependency on the applied compounds and their concentrations, the response of *C*. *glutamicum* also appeared to be prone to cell-to-cell heterogeneity. At intermediate antibiotic cocktail concentrations below the MBC, a fraction of bacteria was not killed but survived exposure, notably without developing heritable resistance to bactericidal concentrations. The development of phenotypically distinct subpopulations is a known phenomenon in mycobacteria [99]. Moreover, the bacterial cell envelope, which represents at least one of the targets of here investigated antibiotics, is a highly dynamic structure [100,101], and a known (myco)bacterial resistance determinant [102,103]. Therefore, stochastically differential cell architecture might cause individual bacterial susceptibility. A potential clinical relevance of this observation needs to be elucidated using mycobacterial infection models in future studies, as well as an evaluation of potential toxicity of compound combinations.

In summary, our study provides an initial characterization of a natural compound cocktail with antibacterial activity, exemplarily demonstrating the potential nature and especially the microbial world continues to offer in the context of antibiotic discovery and drug development. The here immanent potential can be translated into diverse promising solutions to combat infectious diseases. These include combinatorial therapy with two antibiotic compounds [104] where both, synergistic as well as antagonistic effects are discussed as advantageous in the provision of effective and fast treatment of an acute severe infection or the prevention of resistance development [105,106]. Therefore, an in-depth investigation of microbes in competitive ecological niches such as soil should be especially promising for future exploration of novel antibiotic compounds [107] and naturally evolved cocktails including surface active compounds.

### Conclusions

At present, the development of effective antibiotics represents one of the most challenging problems including the identification of novel compounds and formulation. Nature provides not only a rich source of bioactive molecules but also gives hints to promising combinations. To this end, understanding of the ecological contextual function of naturally co-produced antimicrobial secondary metabolites and adoption of or inspiration from these may be a useful strategy for future research on novel antibiotics and antibiotic formulations.

## Supporting information

S1 Fig Disk diffusion assay showing combined effect of prodigiosin together with a control extract from *P*. *putida* with empty expression vector on *C*. *glutamicum*. (PDF)

S2 Fig Disk diffusion assay showing combined effects of prodigiosin and synthetic surfactants on *C*. *glutamicum*. (PDF)

S3 Fig Direct comparison of *C*. *glutamicum* cell morphology during antibiotic exposure at similar growth stages in microfluidic cultivation. (PDF)

S4 Fig Influence of combinatorial treatment with prodigiosin and *N*-myristoyltyrosine on susceptibility of *C*. *glutamicum* after treatment. (PDF)

S5 Fig Sequence alignment of *Serratia* sp. ATCC 39006 putative glycosyltransferase with two putative homologs. (PDF)

S6 Fig Increased prodigiosin production by *S*. *marcescens* in the presence of surfactants in disk diffusion assay. (PDF)

S7 Fig Growth of *C*. *glutamicum* under combined exposure to *N*-myristoyltyrosine and streptomycin. (PDF)

S1 Table Physicochemical properties and inhibitory effects of surfactants applied in this study. (PDF)

S1 Video *C*. *glutamicum* microcolony development in the presence of prodigiosin, *N*-myristoyltyrosine and their combination. From left to right: Control, 1 μg/mL prodigiosin, 15 μg/mL *N*-myristoyltyrosine, combination of 1 μg/mL prodigiosin and 15 μg/mL *N*-myristoyltyrosine.
(WMV)
