# Supplementary material for: Natural biocide cocktails: Combinatorial antibiotic effects of prodigiosin and biosurfactants
Source: PLoS One. 2018 Jul 19;13(7):e0200940. doi: 10.1371/journal.pone.0200940 (PMC6053208; doi:10.1371/journal.pone.0200940)
Supplement: S1 Fig — (PDF) [file pone.0200940.s001.pdf]

Natural biocide cocktails: Combinatorial antibiotic effects of prodigiosin and biosurfactants

Jennifer Hage-Hülsmann, Alexander Grünberger, Stephan Thies, Beatrix Santiago-Schübel, Andreas Sebastian Klein, Jörg Pietruszka, Dennis Binder, Fabienne Hilgers, Andreas Domröse, Thomas Drepper, Dietrich Kohlheyer, Karl-Erich Jaeger, Anita Loeschcke

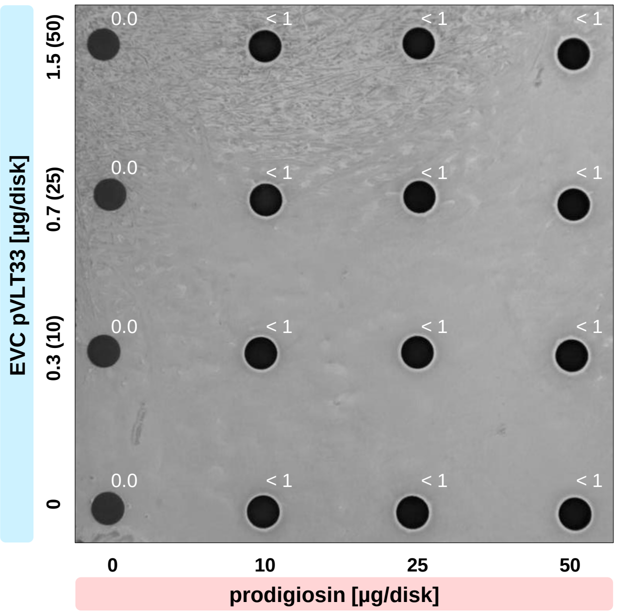

S1 Fig. Disk diffusion assay showing combined effect of prodigiosin together with a control extract from *P. putida* with empty expression vector on *C. glutamicum*. The experiment was conducted to verify specificity of combinatorial effects of prodigiosin and serrawettin W1 which was in this study applied as extract from heterologous production in *P. putida*, and to exclude potential influences of further cellular components in extracts. Combination matrix was composed in a disk diffusion assay on a lawn of *C. glutamicum* using 0, 10, 25, and 50 µg/disk prodigiosin. Instead of extracted serrawettin W1, a suitable control was required. Therefore, the bacterial production procedure was conducted using the empty expression vector (EVC), i.e., pVLT33 (de Lorenzo *et al.* (1993), Gene 123(1):17-24) instead of pVLT-swrW for serrawettin W1 production. Since bacterial production yielded higher extract masses in actual serrawettin W1 production than in empty vector control runs, the difference may be considered as antibiotic-related mass and the other fraction as non-antibiotic cellular components. In case of serrawettin W1, the assumed non-antibiotic fraction was 0.3%. The here applied control extract was thus adjusted to amounts representing the non-antibiotic fraction of 10, 25, and 50 µg serrawettin W1-containing extract. Actually applied amounts are given together with corresponding antibiotic amounts in Fig. 2 of this study in brackets. Reach of inhibition zones from filter disks are indicated [mm].
