# Supplementary material for: Natural biocide cocktails: Combinatorial antibiotic effects of prodigiosin and biosurfactants
Source: PLoS One. 2018 Jul 19;13(7):e0200940. doi: 10.1371/journal.pone.0200940 (PMC6053208; doi:10.1371/journal.pone.0200940)
Supplement: S2 Fig — (PDF) [file pone.0200940.s002.pdf]

Natural biocide cocktails: Combinatorial antibiotic effects of prodigiosin and biosurfactants

Jennifer Hage-Hülsmann, Alexander Grünberger, Stephan Thies, Beatrix Santiago-Schübel, Andreas Sebastian Klein, Jörg Pietruszka, Dennis Binder, Fabienne Hilgers, Andreas Domröse, Thomas Drepper, Dietrich Kohlheyer, Karl-Erich Jaeger, Anita Loeschcke

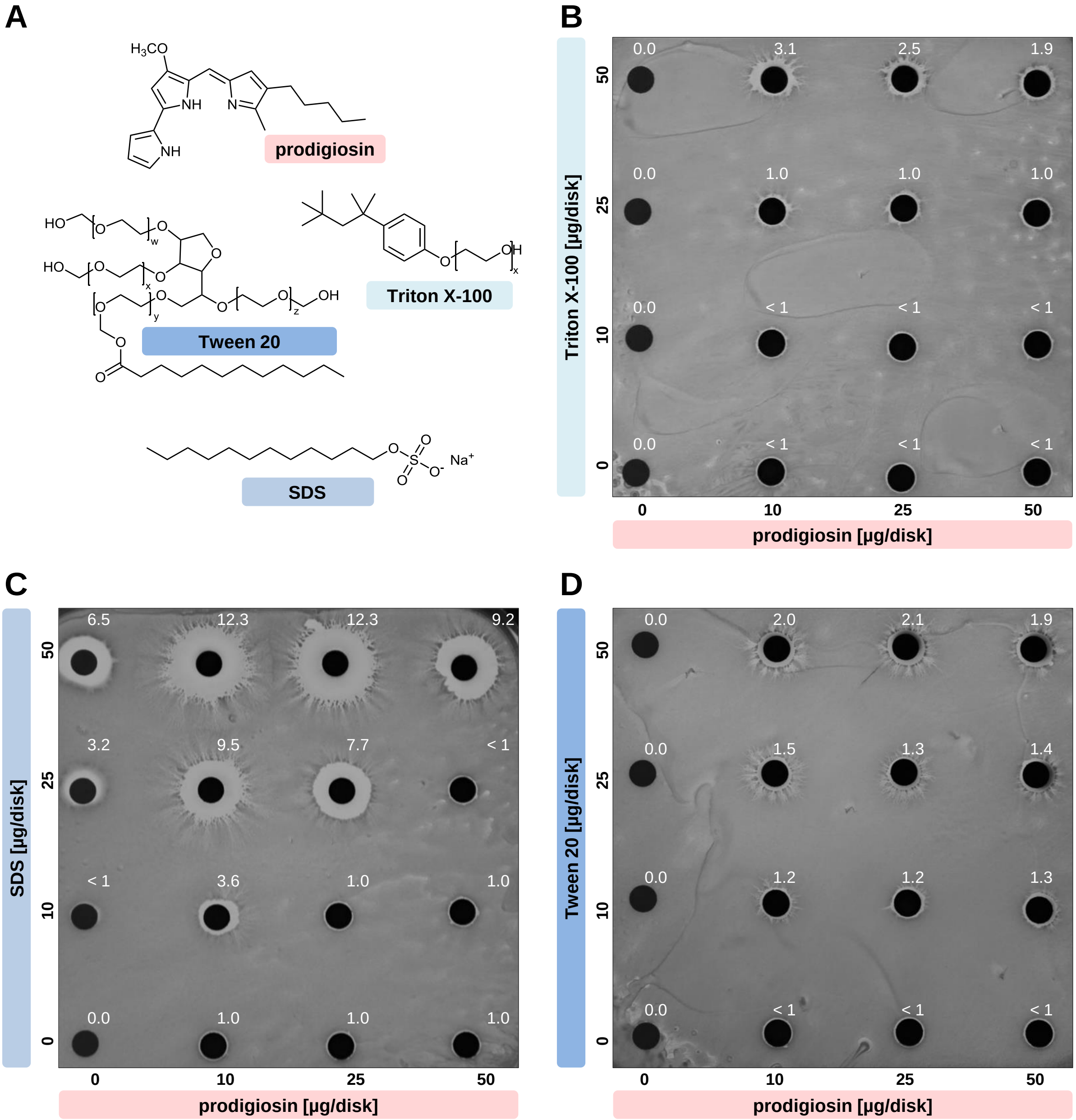

S2 Fig. Disk diffusion assay showing combined effects of prodigiosin and synthetic surfactants on *C. glutamicum*. (A) Applied compounds: Antibacterial pigment prodigiosin and synthetic surfactants Triton X-100 (x = 9-10), SDS and Tween 20 (w + x + y + z = 16). Combination matrices were implemented in disk diffusion assays on a lawn of *C. glutamicum* with compound combinations in series of 0, 10, 25, and 50 µg/disk: **(B)** Combination of prodigiosin and Triton X-100, **(C)** combination of prodigiosin and SDS, **(D)** combination of prodigiosin and Tween 20. Shown photographed results are representative of triplicate experiments, all showing the same tendencies. Reach of inhibition zones from filter disks are indicated [mm].
