# Supplementary material for: Natural biocide cocktails: Combinatorial antibiotic effects of prodigiosin and biosurfactants
Source: PLoS One. 2018 Jul 19;13(7):e0200940. doi: 10.1371/journal.pone.0200940 (PMC6053208; doi:10.1371/journal.pone.0200940)
Supplement: S3 Fig — (PDF) [file pone.0200940.s003.pdf]

Natural biocide cocktails: Combinatorial antibiotic effects of prodigiosin and biosurfactants

Jennifer Hage-Hülsmann, Alexander Grünberger, Stephan Thies, Beatrix Santiago-Schübel, Andreas Sebastian Klein, Jörg Pietruszka, Dennis Binder, Fabienne Hilgers, Andreas Domröse, Thomas Drepper, Dietrich Kohlheyer, Karl-Erich Jaeger, Anita Loeschcke

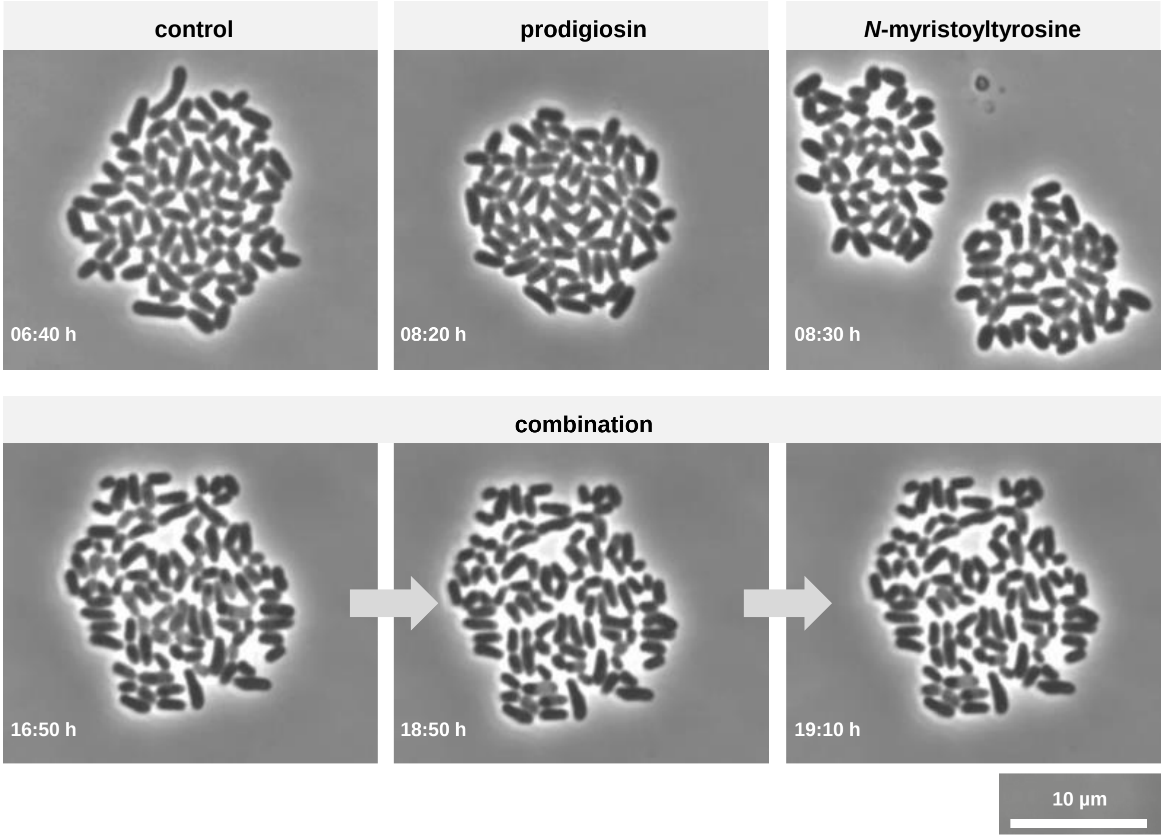

S3 Fig. Direct comparison of *C. glutamicum* cell morphology during antibiotic exposure at similar growth stages in microfluidic cultivation. Extracted images of the S1 Video of microfluidic single cell cultivation show *C. glutamicum* colonies at similar growth stages under different experimental conditions (upper row: control, 1 µg/mL prodigiosin, 15 µg/mL *N*-myristoyltyrosine, lower row: supplementation of 1 µg/mL prodigiosin and 15 µg/mL *N*-myristoyltyrosine in combination). Colonies with similar sizes of approximately 15 µm diameter were chosen for a direct comparison of cell morphology. These colony sizes were reached at the different time points as indicated in each image (in hours after microcolony inoculation). The microcolony in the control chamber reached the chosen size after 06:40 h. Compared to this control, the growth speed of *C. glutamicum* cells was reduced if prodigiosin or *N*-myristoyltyrosine was supplemented, since similar colony areas were reached after 08:20 and after 08:30 h, respectively. In all three cases, these growth stages were transitional and finally resulted in completely filled growth chambers. In contrast, the combinatorial application evoked an even stronger reduced growth speed as the colony only reached a comparable size after 16:50 h. Here, no further colony development was observed (cf. 18:50 and 19:10 h). Cell morphologies in microcolonies treated with the combination moreover differed from control cells and cells which were only exposed to one of the compounds, exhibiting unusual cell shapes and optical properties (brighter grey appearance instead of the usual dark grey), and shrinkage (the decrease of cell sizes becomes apparent by the increase of the interspace between cells). The here described different cell morphologies of *C. glutamicum* cells after combinatorial antibiotic supplementation can be retraced in the S1 Video.
