# Supplementary material for: Natural biocide cocktails: Combinatorial antibiotic effects of prodigiosin and biosurfactants
Source: PLoS One. 2018 Jul 19;13(7):e0200940. doi: 10.1371/journal.pone.0200940 (PMC6053208; doi:10.1371/journal.pone.0200940)
Supplement: S4 Fig — (PDF) [file pone.0200940.s004.pdf]

Natural biocide cocktails: Combinatorial antibiotic effects of prodigiosin and biosurfactants

Jennifer Hage-Hülsmann, Alexander Grünberger, Stephan Thies, Beatrix Santiago-Schübel, Andreas Sebastian Klein, Jörg Pietruszka, Dennis Binder, Fabienne Hilgers, Andreas Domröse, Thomas Drepper, Dietrich Kohlheyer, Karl-Erich Jaeger, Anita Loeschcke

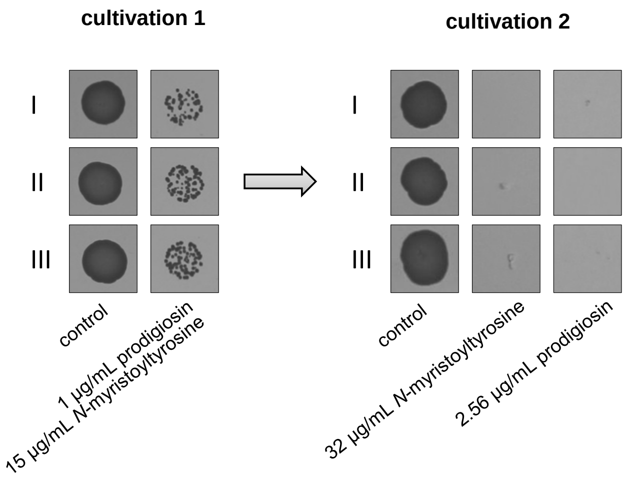

S4 Fig. Influence of combinatorial treatment with prodigiosin and *N*-myristoyltyrosine on susceptibility of *C. glutamicum* after treatment. After treatment with the sub-lethal combination of 1 µg/mL prodigiosin and 15 µg/mL *N*-myristoyltyrosine in small scale liquid cultivation (800 µL), 3 µL samples of diluted culture (1:100) were spotted on agar plates for cultivation in the absence of antibiotics to promote colony formation from surviving cells (left, cultivation 1). To investigate susceptibility of survivors to antibiotics, grown clones were subjected to treatment with previously determined MBC concentration of 2.56 µg/mL prodigiosin or 32 µg/mL *N*-myristoyltyrosine (Fig. 2 in the main manuscript) in liquid cultivation. In the same procedure as before, samples were subsequently cultivated on agar plates in the absence of antibiotics, not giving rise to any survivor colonies – indicative for unchanged susceptibility to MBC concentrations treatment (right, cultivation 2). Independent triplicate experiments are indicated (I, II, III). In both cultivations, cultures were supplemented with the solvent only (ethanol, p.a.) with a final concentration of 3% as a control.
