# Supplementary material for: Natural biocide cocktails: Combinatorial antibiotic effects of prodigiosin and biosurfactants
Source: PLoS One. 2018 Jul 19;13(7):e0200940. doi: 10.1371/journal.pone.0200940 (PMC6053208; doi:10.1371/journal.pone.0200940)
Supplement: S5 Fig — (PDF) [file pone.0200940.s005.pdf]

Natural biocide cocktails: Combinatorial antibiotic effects of prodigiosin and biosurfactants

Jennifer Hage-Hülsmann, Alexander Grünberger, Stephan Thies, Beatrix Santiago-Schübel, Andreas Sebastian Klein, Jörg Pietruszka, Dennis Binder, Fabienne Hilgers, Andreas Domröse, Thomas Drepper, Dietrich Kohlheyer, Karl-Erich Jaeger, Anita Loeschcke

Description	Max score	Total score	Query cover	E-value	Identity	Similarity
rhamnosyltransferase [*Dickeya dianthicola*] WP_024106541.1	539	539	98%	0.0	60%	75%
rhamnosyltransferase subunit B [*Pseudomonas aeruginosa* PAO1] NP_252168.1	220	220	91%	4e-68	31%	50%

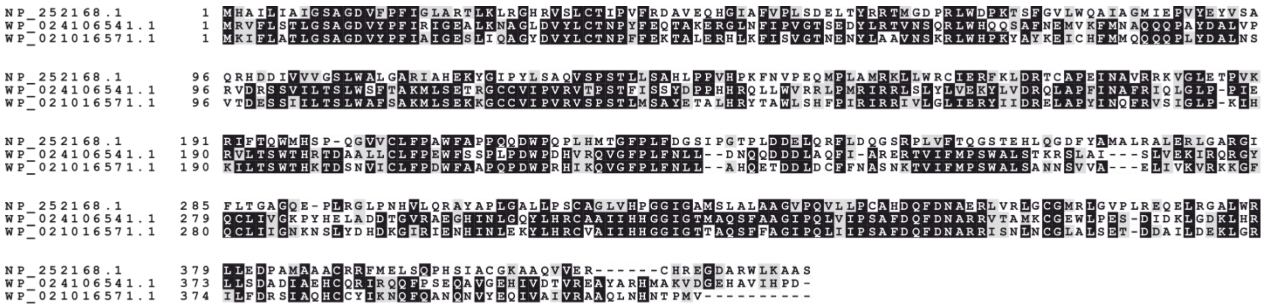

S5 Fig. Sequence alignment of *Serratia* sp. ATCC 39006 putative glycosyltransferase with two putative homologs. The putative glycosyltransferase from *Serratia* sp. ATCC 39006 (NCBI RefSeq WP_021016571.1) was identified by BLASTP similarity search using the known rhamnosyl transferase RhlB from *Pseudomonas aeruginosa* (NCBI RefSeq NP_252168.1) to search for similar sequences within the NCBI protein sequences database limited to *Serratia* sp. ATCC 39006 (Genome at NCBI Genbank Masterentry NZ_AWXH00000000.1).
The sequence of the putative glycosyltransferase from *Serratia* sp. ATCC 39006 was then subjected to NCBI protein sequences database similarity search with BLASTP and yielded the best scoring hit with the sequence of a putative rhamnosyltransferase from *Dickeya dianthicola*. Result table of BLASTP pairwise alignment of the *Serratia* sp. ATCC 39006 glycosyltransferase with the two homologous proteins and Clustal Omega (Sievers *et al*. (2011), doi:10.1038/msb.2011.75) multiple sequence alignment of all three proteins are shown.
