# Supplementary material for: Natural biocide cocktails: Combinatorial antibiotic effects of prodigiosin and biosurfactants
Source: PLoS One. 2018 Jul 19;13(7):e0200940. doi: 10.1371/journal.pone.0200940 (PMC6053208; doi:10.1371/journal.pone.0200940)
Supplement: S6 Fig — (PDF) [file pone.0200940.s006.pdf]

Natural biocide cocktails: Combinatorial antibiotic effects of prodigiosin and biosurfactants

Jennifer Hage-Hülsmann, Alexander Grünberger, Stephan Thies, Beatrix Santiago-Schübel, Andreas Sebastian Klein, Jörg Pietruszka, Dennis Binder, Fabienne Hilgers, Andreas Domröse, Thomas Drepper, Dietrich Kohlheyer, Karl-Erich Jaeger, Anita Loeschcke

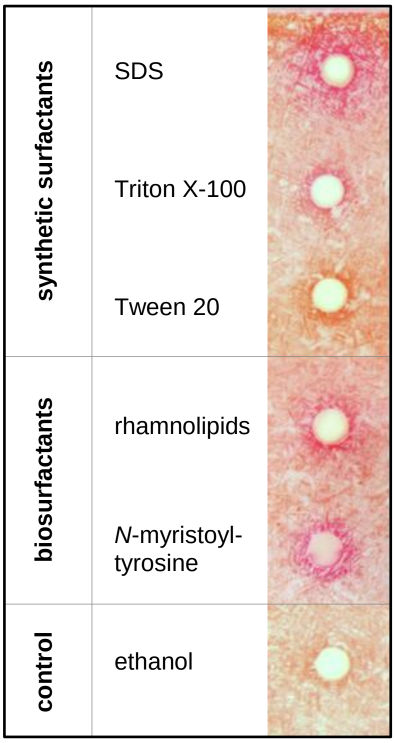

S6 Fig. Increased prodigiosin production by *S. marcescens* in the presence of surfactants in disk diffusion assay. Disk diffusion assays were performed on a lawn of *S. marcescens* with 50 µg/disk of each surfactant at 30 °C. Shown photographed discs are representative of triplicates. Overall contrast of the entire pictures was adjusted to facilitate the optical reception of the effect.
