# Supplementary material for: Natural biocide cocktails: Combinatorial antibiotic effects of prodigiosin and biosurfactants
Source: PLoS One. 2018 Jul 19;13(7):e0200940. doi: 10.1371/journal.pone.0200940 (PMC6053208; doi:10.1371/journal.pone.0200940)
Supplement: S7 Fig — (PDF) [file pone.0200940.s007.pdf]

Natural biocide cocktails: Combinatorial antibiotic effects of prodigiosin and biosurfactants

Jennifer Hage-Hülsmann, Alexander Grünberger, Stephan Thies, Beatrix Santiago-Schübel, Andreas Sebastian Klein, Jörg Pietruszka, Dennis Binder, Fabienne Hilgers, Andreas Domröse, Thomas Drepper, Dietrich Kohlheyer, Karl-Erich Jaeger, Anita Loeschcke

cell density [OD_{650}] in liquid medium under exposure

µg/mL *N*-myristoyltyrosine \ µg/mL streptomycin	0	0.25	0.5	1	2	4	8	16
32	0.04 ± 0.01	0.04 ± 0.01	0.05 ± 0.00	0.03 ± 0.01	0.03 ± 0.00	0.05 ± 0.02	0.03 ± 0.00	0.03 ± 0.00
16	3.12 ± 0.25	0.02 ± 0.01	0.03 ± 0.01	0.02 ± 0.00	0.02 ± 0.00	0.03 ± 0.01	0.02 ± 0.01	0.01 ± 0.01
8	3.89 ± 0.07	0.17 ± 0.04	0.06 ± 0.01	0.05 ± 0.01	0.04 ± 0.01	0.09 ± 0.02	0.05 ± 0.01	0.04 ± 0.01
4	4.18 ± 0.05	3.17 ± 0.24	0.17 ± 0.36	0.10 ± 0.01	0.06 ± 0.02	0.09 ± 0.01	0.08 ± 0.02	0.06 ± 0.01
2	4.34 ± 0.07	3.59 ± 0.25	2.53 ± 0.17	0.26 ± 0.01	0.09 ± 0.00	0.06 ± 0.00	0.05 ± 0.00	0.07 ± 0.02
0	4.32 ± 0.07	3.62 ± 0.29	3.31 ± 0.09	1.67 ± 0.13	0.25 ± 0.01	0.12 ± 0.02	0.06 ± 0.01	0.04 ± 0.01

color category according to OD_{650}	cell growth
< 0.06	no growth
≥ 0.06	strongly impaired
≥ 1.00	moderately impaired
≥ 4.10	indistinguishable from control

S7 Fig. Growth of *C. glutamicum* under combined exposure to *N*-myristoyltyrosine and streptomycin. Liquid cultures (volume 800 µL) of *C. glutamicum* were supplemented with antibiotic compounds in a checkerboard matrix individually and in combinations in two-fold dilutions. Turbidities of cultures were measured at 650 nm after incubation for 20 h at 30 °C and constant agitation. Grey: Turbidities up to 0.06, defined as no growth. Different shades of blue: Strongly impaired growth with turbidities from 0.06 to 1.00, moderately impaired growth with turbidities from 1.00 to 4.10, and unimpaired growth with turbidities above 4.10. Values represent mean results from three independent measurements and the respective standard deviation.
