# Supplementary material for: Natural biocide cocktails: Combinatorial antibiotic effects of prodigiosin and biosurfactants
Source: PLoS One. 2018 Jul 19;13(7):e0200940. doi: 10.1371/journal.pone.0200940 (PMC6053208; doi:10.1371/journal.pone.0200940)
Supplement: S1 Table — (PDF) [file pone.0200940.s008.pdf]

Natural biocide cocktails: Combinatorial antibiotic effects of prodigiosin and biosurfactants

Jennifer Hage-Hülsmann, Alexander Grünberger, Stephan Thies, Beatrix Santiago-Schübel, Andreas Sebastian Klein, Jörg Pietruszka, Dennis Binder, Fabienne Hilgers, Andreas Domröse, Thomas Drepper, Dietrich Kohlheyer, Karl-Erich Jaeger, Anita Loeschcke

S1 Table. Physicochemical properties and inhibitory effects of surfactants applied in this study.

surfactant[1]	type	molecular weight [Da][2]	CMC[3]	compound applied in assay[4]	observed effects[5]
**synthetic surfactants**					
Triton X-100 (octyl phenol ethoxylate)	non-ionic	625	0.2-0.9 mM[a] 0.012-0.056% (in water)	commercial	no inhibition alone, synergistic inhibition with prodigiosin (concentration-dependent)
SDS (sodium dodecyl sulfate)	anionic	288	7-10 mM[a] 0.2-0.29% (in water)	commercial	inhibition alone, synergistic inhibition with prodigiosin (concentration-dependent)
Tween 20 (polysorbate 20)	non-ionic	1228	0.06 mM[a] 0.0072% (in water)	commercial	no inhibition alone, synergistic inhibition with prodigiosin (concentration-independent)
**biosurfactants**					
serrawettin W1 (*Serratia marcescens*)	non-ionic	515 (C10, C10)	unknown	raw extract from *P. putida*	no inhibition alone, synergistic inhibition with prodigiosin (concentration-dependent)
rhamnolipids (*Pseudomonas aeruginosa*)	anionic	679 (dirhamnolipid, C10, C10)	0.0005-0.02%[b] (dependent on conditions and congener composition)	commercial (from *P. aeruginosa*)	inhibition alone, synergistic inhibition with prodigiosin (concentration-dependent)
N-myristoyl-tyrosine (unknown)	anionic	392	2.9 mM[c] 0.114% (in 0.1 M NaOH)	chemical synthesis	inhibition alone, synergistic inhibition with prodigiosin (concentration-dependent)

[1] compound with chemical designation (synthetic surfactants) or natural producer organism (biosurfactants).
[2] Triton X-100 contains 9-10 ethylene oxide units per molecule (see also S3 Fig.); MW is given as an average. Biosurfactants consist of several congeners differing in the hydroxyl fatty acids and in case of rhamnolipids in the number of sugar moieties; MW is given for one prominent congener and chain lengths are indicated.
[3] CMCs (critical micellar concentrations) are listed as determined in other studies; validity for experiments of the present study is limited as they are much dependent on the exact assay conditions. [a] Sigma Aldrich "detergent_selection_table", [b] [57], [c] [44].
[4] source of the compounds for their application in assays of the present study.
[5] summarized results from disk diffusion assays in a checkerboard matrix together with prodigiosin using *C. glutamicum* as model bacterium.
